# *Eurosurveillance*: 25 years of public health impact

**DOI:** 10.2807/1560-7917.ES.2021.26.49.211209ed

**Published:** 2021-12-09

**Authors:** Eurosurveillance editorial team

**Affiliations:** 1 European Centre for Disease Prevention and Control (ECDC), Stockholm, Sweden

The scientific community’s interest in communicable diseases gained momentum during the last two decades of the 20th century, following the emergence of HIV and the re-emergence of (drug-resistant) tuberculosis, as well as a general increase in mortality and morbidity from infectious diseases [1]. In the mid-1990s, a number of initiatives were established in Europe that comprised, among others, the European Union (EU) dedicated surveillance networks and field epidemiology training programmes. In this context, there was an evident need for an exchange of information on results from the surveillance activities, on emerging public health threats and on the detection and response to outbreaks to support public health action and decision-making.

The monthly *Eurosurveillance* journal, created in 1995 and fully operational since 1996, was set up as a platform for outputs from the EU surveillance networks and *to disseminate national experiences that other countries of the EU may find useful* [2]. To complement the monthly journal, the *Eurosurveillance* weekly bulletin was created in 1996. It contained short edited news-like articles on outbreaks and important infectious disease events globally, reviewed by editors and their colleagues, with a European focus. The weekly and monthly editions were merged into today’s *Eurosurveillance* in 2008 to capitalise on the strengths of the two journals [3]. In particular the short articles have, over time, developed into a source of sound scientific information that has helped shape public health response and policies at local, national and international level.

Being at the forefront of establishing rapid information exchange as an accepted means of peer-reviewed, scholarly communication has been an important pillar of the journal’s impact. Over the years, a small but dedicated team – supported by a pan-European editorial board and working closely with many scientists and public health experts – has established the journal firmly among the prestigious journals in the field of infectious diseases. *Eurosurveillance* has ranked among the top 10 in its category for 10 years in the Journal Citation Reports impact factor and other metrics (SCImago Journal Rank, Scopus CiteScore, Google Scholar) have also been good. We take the combination of anecdotal/narrative evidence (see examples in our anniversary collection), the feedback from formal evaluations [4] and good metrics as an indication of the journal’s public health impact during its 25 years of existence.

An increasing number of scholarly journals have implemented fast-tracked, peer-reviewed publishing. Together with the increased use and acceptance of preprint servers, this has facilitated important early information exchange and informed public health decision-making. The urgent need for evidence during the ongoing coronavirus disease (COVID-19) pandemic has accelerated such initiatives and led to profound changes in scholarly publishing. We continue to follow these developments closely, weighing which would be of benefit to our readers and contributors and which we would approach more conservatively.

In 2021, *Eurosurveillance* has celebrated its 25th anniversary, prompting us to look backwards as well as forwards. Looking to the future, we have identified a number of areas that will guide our operations in the coming years, while we will of course remain a fully open-access and non-profit journal. Aside from the speed of our rapid communications, quality and correctness of content and clear public health messages have been our focus. We believe and have evidence to suggest that we have gained the trust of our audience and that articles published in the journal are considered to provide sound and reliable scientific information [4,5]. Quality control (peer review, evaluation and editing), however, has come with a certain cost with regards to the time to publication for longer articles. In the future, both speed and authoritativeness are key features of the journal that we wish to preserve and strengthen further. We also plan to work with our authors to tease out clear public health messages even more than in the past. The application of classical infectious disease epidemiological methods, in combination with public health microbiology in outbreak investigations and surveillance, has increasingly been established as a principle and has been reflected in many articles over the years. The growing complexity of our environment and the continuous development of novel methods in other disciplines that can be applied for infectious disease prevention and control, necessitates an even stronger focus on interdisciplinary aspects in the articles we select for review. Taking a gradual approach, we will address this through annual themes and subthemes that should help guide the selection of attractive and relevant articles for our audience.

We remain vigilant to new developments in publishing and we are attentive to our core mission. We will carry on supporting public health practice and policymaking through sharing knowledge and authoritative evidence. Sustainability and diversity will be important guiding principles for our work. They should help us make an impact on public health also in the future and, as in the past, we count on the support of our peer-reviewers, board members and colleagues to reach this goal.
